# Acylation Stimulating Protein, Complement C3 and Lipid Metabolism in Ketosis-Prone Diabetic Subjects

**DOI:** 10.1371/journal.pone.0109237

**Published:** 2014-10-02

**Authors:** Yan Liu, Priyanka Gupta, Marc Lapointe, Thewjitcharoen Yotsapon, Sunthornyothin Sarat, Katherine Cianflone

**Affiliations:** 1 Centre de Recherche de l’Institut Universitaire de Cardiologie & Pneumologie de Québec, Université Laval, Québec, Canada; 2 Department of Pediatrics, Tongji Hospital, HuaZhong University of Science and Technology, Wuhan, Hubei, P. R. China; 3 Division of Endocrinology and Metabolism, Department of Medicine, Faculty of Medicine, Chulalongkorn University, Bangkok, Thailand; Steno Diabetes Center, Denmark

## Abstract

**Background:**

Ketosis-prone diabetes (KPDM) is new-onset diabetic ketoacidosis without precipitating factors in non-type 1 diabetic patients; after management, some are withdrawn from exogenous insulin, although determining factors remain unclear.

**Methods:**

Twenty KPDM patients and twelve type 1 diabetic patients (T1DM), evaluated at baseline, 12 and 24 months with/without insulin maintenance underwent a standardized mixed-meal tolerance test (MMTT) for 2 h.

**Results:**

At baseline, triglyceride and C3 were higher during MMTT in KPDM vs. T1DM (p<0.0001) with no differences in non-esterified fatty acids (NEFA) while Acylation Stimulating Protein (ASP) tended to be higher. Within 12 months, 11 KPDM were withdrawn from insulin treatment (KPDM-ins), while 9 were maintained (KPDM+ins). NEFA was lower in KPDM-ins vs. KPDM+ins at baseline (p = 0.0006), 12 months (p<0.0001) and 24 months (p<0.0001) during MMTT. NEFA in KPDM-ins decreased over 30–120 minutes (p<0.05), but not in KPDM+ins. Overall, C3 was higher in KPDM-ins vs KPDM+ins at 12 months (p = 0.0081) and 24 months (p = 0.0019), while ASP was lower at baseline (p = 0.0024) and 12 months (p = 0.0281), with a decrease in ASP/C3 ratio.

**Conclusions:**

Notwithstanding greater adiposity in KPDM-ins, greater NEFA decreases and lower ASP levels during MMTT suggest better insulin and ASP sensitivity in these patients.

## Introduction

Diabetic ketoacidosis (DKA) is the most serious hyperglycemic emergency in patients with diabetes. It has long been assumed that DKA is a key clinical feature of type 1 diabetes (T1DM) [1]. However, in the past 2 decades, an increasing number of ketoacidosis cases without precipitating causes have also been reported in children and adults with T2DM [2], [3]. These subjects present with mixed features of both type 1 and type 2 diabetes, and this has been recently referred to as ketosis-prone diabetes mellitus (KPDM) [4], [5]. KPDM has been reported primarily in Africans and African-Americans [5], [6]. However, during the past decade, it has been increasingly recognized and reported in other ethnic groups (such as Chinese, Korean, Hispanic and Caucasian) [7], [8], [9].

KPDM is characterized by presentation with DKA or unprovoked ketosis in persons who do not fit the traditional categories of type 1 or type 2 diabetes [10]. It is unclear why these patients are more susceptible to DKA. Current knowledge suggests that development of an acute reduction in insulin production, coupled to the underlying insulin resistance, might precipitate a ketotic episode [3].

Most patients with KPDM are overweight/obese, middle-aged, and have a low prevalence of autoimmune markers. At presentation of DKA, they fulfill the same biochemical criteria for ketoacidosis as those with T1DM. However, after aggressive diabetes management, their β-cell function can be partially or fully restored. Therefore, a subset of KPDM patients can safely discontinue exogenous insulin and achieve near-normoglycemic remission for months to several years [6], [8], [11]. However, the pathophysiologic mechanisms involved in its cause or remission remain unknown.

In recent years, several studies have reported clinical characteristics and factors associated with insulin discontinuation in patients with KPDM [6], [8], [11]. In KPDM patients, the best predictors of insulin discontinuation are (i) presenting with new-onset diabetes and (ii) maintaining higher β-cell function reserves (as measured by the C-peptide-to-glucose ratio) [11]. Overweight/obesity is one important characteristic of KPDM. As patients with KPDM develop progressive increases in body weight both before the onset of initial DKA and before the onset of ketotic relapse, obesity also seems to predispose to β-cell failure in these subjects [6]. Whether it is an important predictor of insulin discontinuation in KPDM is still controversial. Maldonado et al [11] showed that there was no difference in mean BMI between insulin-withdrawal and insulin-maintenance KPDM patients. However, another study demonstrated that KPDM patients with higher BMI were non-insulin-dependent and had greater success in discontinuing insulin therapy [6]. It is not clear whether there exists any correlation in lipid metabolism disturbances and insulin dependence in KPDM patients.

Links between complement component 3 (C3) and acylation stimulating protein (ASP) with diabetes and well-recognized risk factors such as lipids and glucose/insulin have already been studied in detail. It is well known that human adipose tissue can synthesize and secrete C3 [12]. Engstrom et al [13] have demonstrated that C3 is a powerful risk factor in the development of diabetes in men aged 38–50 years and serum C3 strongly correlates with insulin resistance [14]. The interaction of complement C3, factor B and the enzyme adipsin generates C3 convertase (C3bBb), which cleaves C3 into C3a and C3b. Carboxypeptidases then cleave the terminal arginine from C3a to produce ASP (C3adesArg) [12]. As a lipogenic factor, ASP is implicated in glucose and lipid metabolism and contributes to the increase in fat mass and weight gain [15]. Fasting ASP is predictive of postprandial triglyceride (TG) clearance [16]. C3 and ASP have also been shown to be increased in diabetes. As diabetes is often associated with obesity, this may be a confounding factor [11].

We hypothesized that dysfunctional regulation of C3, ASP and lipid metabolism might exist in KPDM patients and also might relate to their impaired β-cell function. Further, we propose that these parameters may contribute to the potential for insulin withdrawal in KPDM patients. In this study, we determine the changes in lipid parameters, C3 and ASP, both fasting and following a mixed meal, with evaluation over 2 years following the ketotic incident.

## Materials and Methods

### Subjects

Twenty ketosis-prone diabetic patients (KPDM) were recruited from the King Chulalongkorn Memorial Hospital of Thailand. For control subjects, 12 type 1 diabetic patients were recruited. These type 1 diabetic subjects were characterized as age ≤30 years, BMI ≤23 kg/m^2^ and a requirement of insulin for life. KPDM was identified as new-onset diabetes without precipitating illness (infection, stress), with the presence of strong ketosis or DKA [6]. The study was approved by the Institutional Review Boards at King Chulalongkorn Memorial Hospital, Bangkok, Thailand and all patients provided written consent. The subjects were followed over a 2-year period, during which time exogenous insulin was discontinued following the ketotic episode (between 1–12 months after) at the discretion of the treating physician. All of these patients were given Metformin as an oral agent to prevent the relapse of DKA. During the time frame of the follow-up period (24 months), no patients relapsed into hyperglycemia. Relapse was defined as the reoccurrence of ketosis or fasting plasma glucose ≥250 mg/dL. KPDM patients were divided into two groups (insulin withdrawal and insulin maintenance) during data analysis at the end of the follow-up period for all subjects.

### Mixed-meal tolerance test (MMTT)

After resolution of DKA within 2 weeks, MMTT were conducted in all patients at baseline, 12 and 24 months. Following an overnight fast (at least 8 h), and withholding of insulin injection or oral agents (at least 12 hours), the subjects were given a mixed meal consisting of 65% carbohydrates, 21% protein and 14% fat (6 ml/kg of Ensure up to 360 ml). The total calories per serving were less than 360 kcal. Blood samples were taken at 0, 30, 60, 90 and 120 minutes after the meal.

### Blood sample measurements

Plasma glucose, C-peptide and HbA1C were measured at King Chulalongkorn Memorial Hospital of Thailand. Serum was stored in aliquots at −70°C until analysis. Samples were shipped to the research center of the University Institute of Cardiology and Respirology of Quebec (CRIUCPQ) for additional analysis. Serum triglyceride and non-esterified fatty acids (NEFA) were measured by colorimetric enzymatic kits as follows: serum triglyceride (Roche Diagnostics, Indianapolis, IN, USA), NEFA (Wako Pure Chemicals, Richmond, VA, USA). Complement C3 was measured by immunoturbidimetry (Kamiya Biochemical Company, Seattle, WA, USA). Human ASP was measured by in-house sandwich ELISA [17].

### Statistical Analysis

All results are expressed as mean ± S.D. in the tables and text and as mean ± S.E.M. in the figures as indicated. Fasting baseline data values were compared between KPDM and T1DM patients using unpaired Student’s t-test and gender and new onset status between groups were compared using Chi-square test. Comparisons across different times in KPDM with insulin withdrawal (KPDM-ins) or KPDM with insulin maintenance (KPDM+ins) were analyzed by one-way ANOVA followed by Newman-Keuls post-hoc test. Comparison between the two groups at different time points were analyzed by two-way ANOVA followed by post-hoc tests. Statistical analysis was performed with GraphPad Prism5 (San Diego, CA, USA). Significance was set at P<0.05, where NS indicates ‘not significant’.

## Results

### Clinical and biochemical characteristics of KPDM and T1DM patients at baseline

The clinical and laboratory characteristics of the patients with KPDM and T1DM are shown in Table 1. The mean age and body mass index (BMI) of KPDM patients at time of diagnosis was higher than that of T1DM patients. While fasting C-peptide levels in KPDM were higher than in T1DM there was no difference in fasting glucose and HbA1C. Concentrations of triglyceride and C3 were higher in KPDM compared to T1DM patients while non-esterified fatty acids (NEFA), ASP and ASP/C3 ratio tended to be higher (but were not significant).

**Table 1:** Clinical and biochemical characteristic of KPDM and T1DM at baseline.

	KPDM	T1DM	P value
**n**	20	12	
**Age (years)**	38.8±11.5	26.7±10.3	P = 0.005
**Gender (M/F)**	14/6	5/7	NS
**BMI (kg/m^2^)**	28.9±5.56	21.8±3.77	P = 0.0005
**New Onset**	19/20	3/12	P0.0001
**Glucose (mmol/L)**	7.64±2.46	8.33±2.82	NS
**C-Peptide (ng/mL)**	0.910±0.674	0.233±0.192	P = 0.0021
**HbA1C (%)**	11.5±3.43	10.6±2.19	NS
**TG (mmol/L)**	1.66±1.01	0.808±0.304	P = 0.0152
**NEFA (mmol/L)**	0.942±0.682	0.657±0.294	NS
**C3 (g/L)**	1.62±0.24	1.20±0.248	P = 0.0002
**ASP (nmol/L)**	275±174	166±106	P = 0.059
**ASP/C3**	156±92.8	135±105	NS

All values are fasting values for KPDM and T1DM and are expressed as mean ± SD, where significance was evaluated by 2-tailed t test.

### Circulating concentrations of adipokines during 2-h MMTT in KPDM and T1DM patients at baseline

**Figure 1:**
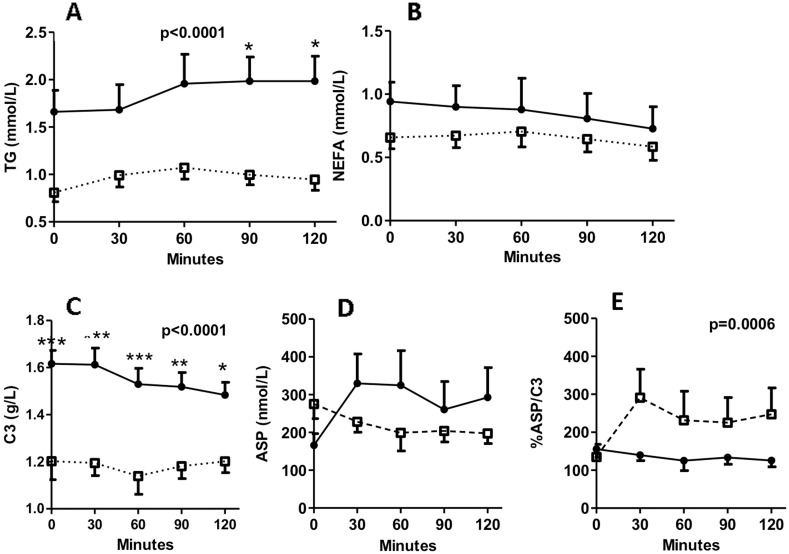
Circulating concentrations of lipids, C3 and ASP. KPDM patients (black circles, solid line) and T1DM patients (white squares, dotted line) at baseline ingested a standard mixed meal tolerance test, and results are presented for triglyceride (TG) (A), NEFA (B), complement C3 (C), ASP (D), and %ASP/C3 (E) measured from 0 to 120 minutes. Results are expressed as mean ± S.E.M; data are analyzed by two-way ANOVA with Bonferroni post-tests as described in methods, where *P<0.05, **P<0.01 and ***P<0.001 and statistical comparisons refer to KPDM vs T1DM at the same time point, unless otherwise indicated.

Following an overnight fast (at least 8 h), and withholding of insulin injection or oral agents (at least 12 hours), mixed meal tolerance tests (MMTT), consisting of 65% carbohydrate, 21% protein, and 14% fat, were ingested at baseline in both groups. During the MMTT, triglyceride levels were elevated at 90 and 120 min in KPDM patients when compared with T1DM patients (Figure 1A). As well, C3 concentrations were higher at fasting and postprandially in KPDM compared to T1DM subjects (Figure 1C). However, there was no significant difference in NEFA and ASP levels between the two groups during MMTT, and no significant change over time for either NEFA or ASP (Figure 1B and 1D). Overall, the lipid parameters triglyceride and NEFA as well as the adipokines C3 and ASP remained relatively constant throughout the MMTT, which contained primarily carbohydrates with no significant change (Figure 1A–D, pNS ANOVA). Although there was no difference in fasting ASP/C3 ratio between the two groups, the mean value of ASP/C3 during the MMTT was significant lower in KPDM than that in T1DM patients (P = 0.0006, two-way ANOVA, Figure 1E).

### Clinical and biochemical follow-up in KPDM patients

The subjects were followed over a 2-year period, during which time exogenous insulin was discontinued in some of the patients at the discretion of the treating physician between 1–12 months following the ketotic episode. In the remaining subjects, exogenous insulin was still needed for the treatment of diabetes during the two years of the study (insulin maintenance = KPDM+ins, n = 9 patients). In those with insulin withdrawal (insulin withdrawal =  KPDM-ins, n = 11 subjects), exogenous insulin was safely discontinued as per the recommendation of their physicians. 6 subjects stopped taking insulin within 4 months, while 5 other patients ceased insulin between months 4 and 12. Average time of insulin withdrawal was 5.1±3.3 months, n = 11). Near normo-glycemic remission was obtained with oral hypoglycemic agents (metformin) in the patients with insulin withdrawal.

Fasting values for KPDM+ins and KPDM-ins patients are shown in Table 2 for the two-year period. Body mass index (BMI) was higher in insulin-withdrawal group than that in insulin-maintenance KPDM. Fasting glucose was significantly lower in insulin-withdrawal subjects than in insulin-maintenance subjects. Fasting C-peptide levels were significantly elevated in insulin-withdrawal compared to insulin-maintenance group. There were no significant differences in fasting HbA1C, triglyceride, NEFA, C3, ASP and ASP/C3 ratio between the two groups.

**Table 2:** Comparions of clinical and biochemical characteristics during follow-up between KPDM with insulin withdrawal and KPDM with insulin maintenance.

	Insulinmaintenance0 month	Insulinmaintenance12 month	Insulinmaintenance24 month	One-wayANOVA	Insulinwithdrawal0 month	Insulinwithdrawal12 month	Insulinwithdrawal24 month	One-wayANOVA	Maintance vs WithdrawTwo-way ANOVA a
**BMI** **(kg/m2)**	26.4±5.44	26.1±5.03	25.9±5.06	NS	31.0±4.98	28.7±3.92	28.0±2.81	NS	**P = 0.0180**
**Glucose** **(mmol/L)**	8.51±2.76	7.723±2.941	8.34±2.40	NS	6.92±2.03	6.05±1.04	6.00±1.04	NS	**P = 0.0025**
**c-peptide** **(ng/mL)**	0.578±0.806	0.522±0.606	0.678±0.661	NS	1.18±0.405	2.23±1.10	1.87±0.475	**P = 0.0116**	**P<0.0001**
**HbA1C (%)**	9.47±2.09	8.61±2.11	8.34±1.62	NS	13.1±3.51	6.46±0.818	6.29±0.842	**P<0.0001**	NS
**TG** **(mmol/L)**	1.77±1.46	1.51±0.706	1.44±0.799	NS	1.57±0.477	1.51±0.614	1.19±0.347	NS	NS
**NEFA** **(mmol/L)**	1.09±0.950	0.570±0.165	0.815±0.457	NS	0.820±0.353	0.644±0.232	0.751±0.283	NS	NS
**C3 (g/L)**	1.55±0.292	1.53±0.252	1.45±0.323	NS	1.66±0.208	1.62±0.377	1.69±0.263	NS	NS
**ASP** **(nmol/L)**	330±200	244±145	301±258	NS	230±144	207±134	316±287	NS	NS
**ASP/C3**	188±120	155±77.2	170±137	NS	135±69.1	141±121	207±217	NS	NS

All values are fasting values for KPDM and T1DM at baseline, 12 and 24 months of follow-up. Results are expressed as mean ± SD, where significance was evaluated by 2-way ANOVA or 1-way ANOVA.

For all of these parameters, there was no significant difference over the 24-month time period (baseline, 12 and 24 months) in the insulin-maintenance patients. The levels of fasting C-peptide were increased at 12 months compared to baseline (P<0.05) in the insulin withdrawal group, but tended to drop by 24 months. The concentration of HbA1C was decreased at 12 months and 24 months compared to baseline in the insulin-withdrawal group. There were no significant differences in the other parameters in the insulin-withdrawal patients.

### Circulating concentrations of triglyceride during 2-h MMTT in insulin-maintenance and insulin-withdrawal groups at baseline, 12 months and 24 months

**Figure 2:**
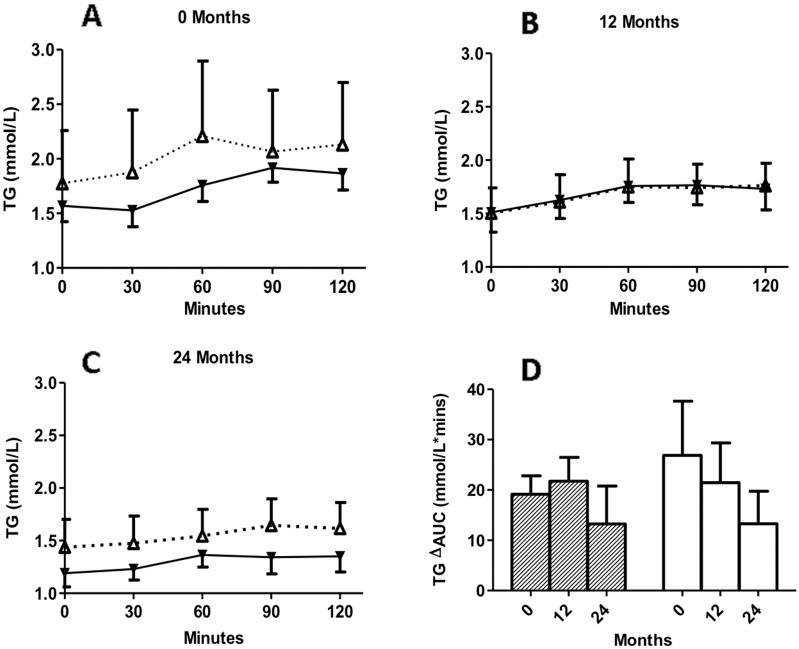
Circulating concentrations of TG. KPDM with insulin maintenance (white triangles, dotted line) and KPDM with insulin withdrawal (black triangles, solid line) were evaluated at baseline and follow-up. Following a standard mixed meal, TG were measured from 0 to 120 minutes at baseline (A), 12 months (B), and 24 months (C). In panel D, results are given for calculated change in area-under-the-curve (ΔAUC) for KPDM with insulin maintenance (white bars) and KPDM with insulin withdrawal (hatched bars) at the indicated times. Results are expressed as mean ± S.E.M; significant changes were evaluated by two-way ANOVA with Bonferroni post-tests as described in methods.

Fasting and postprandial triglyceride concentrations were measured and compared for both time (during MMTT) and group differences during the 3 testing periods. Not surprisingly, given the low fat content of the MMTT, there was little change in triglyceride during the postprandial period (0–120 minutes), and there was no difference between groups. (Figure 2-A–B–C).

The postprandial change in triglyceride during the MMTT was calculated by area-under-the-curve (TG ΔAUC), as shown for the 3 time periods (baseline, 12 and 24 months, Figure 2D). During the 2-year follow-up period, the overall TG ΔAUC tended to decrease in the two groups.

### Circulating concentrations of NEFA during 2-h MMTT in insulin maintenance and insulin withdrawal groups at baseline, 12 months and 24 months

**Figure 3:**
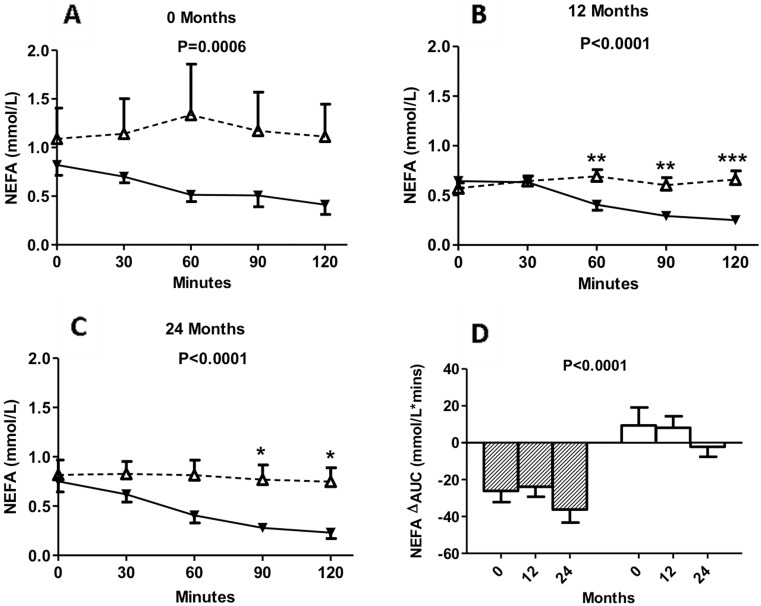
Circulating concentrations of NEFA. KPDM patients with insulin maintenance (white triangles, dotted line) and KPDM patients with insulin withdrawal (black triangle, solid line) were evaluated at baseline and follow-up. Following a standard mixed meal, NEFA were measured from 0 to 120 minutes at baseline (A), 12 months (B), and 24 months (C). In panel D, results are given for calculated change in area-under-the-curve (ΔAUC) for KPDM with insulin maintenance (white bars) and KPDM with insulin withdrawal patients (hatched bars) at the indicated times. Results are expressed as mean ± S.E.M; data are analyzed by two-way ANOVA with Bonferroni post-tests as described in methods, where *P<0.05, **P<0.01 and ***P<0.001 and comparisons refer to KPDM with insulin maintenance vs KPDM with insulin withdrawal at the same time point, unless otherwise indicated.

During the MMTT, NEFA levels in the insulin-withdrawal group (KPDM-ins) fell, reaching a minimum at 120 minutes, with significant differences at all postprandial time points vs. fasting. However, there was no significant drop during the MMTT in the insulin maintenance group (Figure 3A, 3B and 3C), and the NEFA levels in the insulin-maintenance patients remained significantly higher than those in the insulin-withdrawal patients at all time points (Figure 3-A–B–C).

The postprandial decreases in NEFA over time (NEFA ΔAUC), as shown in Figure 3D, were significantly greater in the insulin-withdrawal group during the 2-year follow-up compared to the insulin-maintenance group at all assessment points (baseline, 12 and 24 months).

### Circulating concentrations of C3 during 2-h MMTT in insulin maintenance and insulin withdrawal groups at baseline, 12 months and 24 months

**Figure 4:**
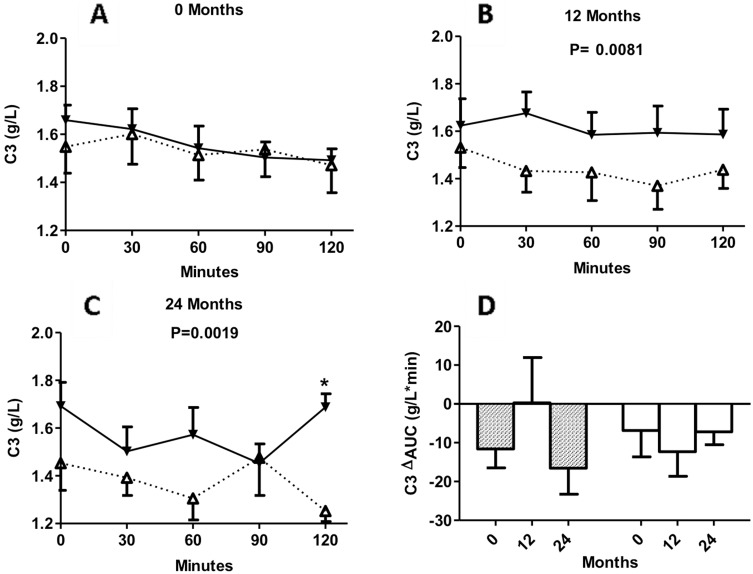
Circulating concentrations of C3. KPDM subjects with insulin maintenance (white triangles, dotted line) and KPDM subjects with insulin withdrawal (black triangles, solid line) were evaluated at baseline and follow-up. Following a standard mixed meal, C3 was measured from 0 to 120 minutes at baseline (A), 12 months (B), and 24 months (C). In panel D, results are given for calculated area-under-the-curve (ΔAUC) for insulin maintenance (white bars) and insulin withdrawal patients (hatched bars) at the indicated times. Results are expressed as mean ± S.E.M; data are analyzed by two-way ANOVA with Bonferroni post-tests as described in methods, where *P<0.05 and comparisons refer to insulin maintenance vs insulin withdrawal at the same time point, unless otherwise indicated.

The changes in C3 concentrations during the MMTT in both groups are shown in Figure 4A–C. Although there was no significant difference between the two groups during the MMTT at baseline, C3 concentrations were significantly higher in the patients withdrawn from insulin at 12 and 24 months vs. those on insulin-maintenance (Figure 4B and 4C).

There were no significant differences in C3 ΔAUC between the insulin-maintenance and insulin-withdrawal groups, or differences between follow-up time (baseline, 12 and 24 months, Figure 4D).

### Circulating concentrations of ASP during 2-h MMTT in insulin maintenance and insulin withdrawal groups at baseline, 12 months and 24 months

**Figure 5:**
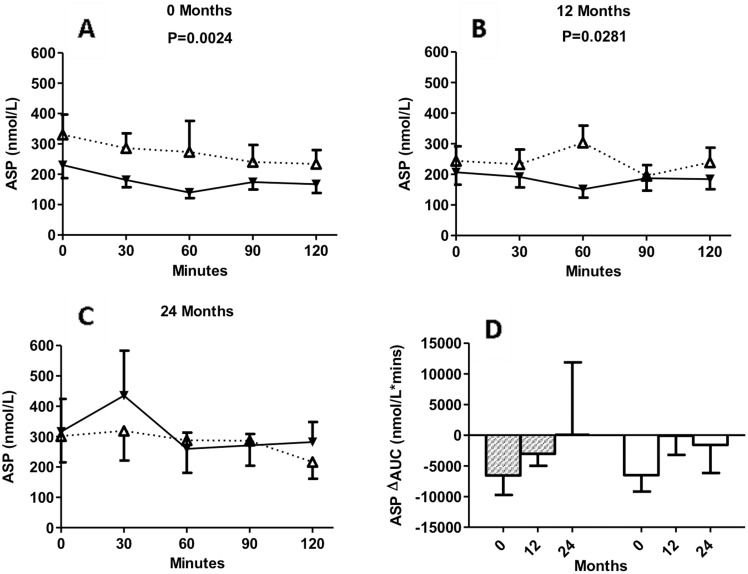
Circulating concentrations of ASP. KPDM subjects with insulin maintenance (white triangles, dotted line) and KPDM subjects with insulin withdrawal (black triangles, solid line) were evaluated at baseline and follow-up. Following a standard mixed meal, ASP was measured from 0 to 120 minutes at baseline (A), 12 months (B), and 24 months (C). In panel D, results are given for calculated area-under-the-curve (ΔAUC) for insulin maintenance (white bars) and insulin withdrawal patients (hatched bars) at the indicated times. Results are expressed as mean ± S.E.M; data are analyzed by two-way ANOVA with Bonferroni post-tests as described in methods.

Both fasting and during the MMTT, baseline and 12 month ASP concentrations were significantly lower in the insulin-withdrawal group than those in insulin-maintenance group (Figure 5A, 5B).

The MMTT change in ASP is given in Figure 5D. While there was no significant difference in ASP ΔAUC between the two groups, to some extent the drop in ASP (ΔAUC) tended to be less at 24 months compared with baseline in both groups.

### Values of %ASP/C3 ratio during 2-h MMTT in insulin maintenance and insulin withdrawal groups at baseline, 12 months and 24 months

**Figure 6:**
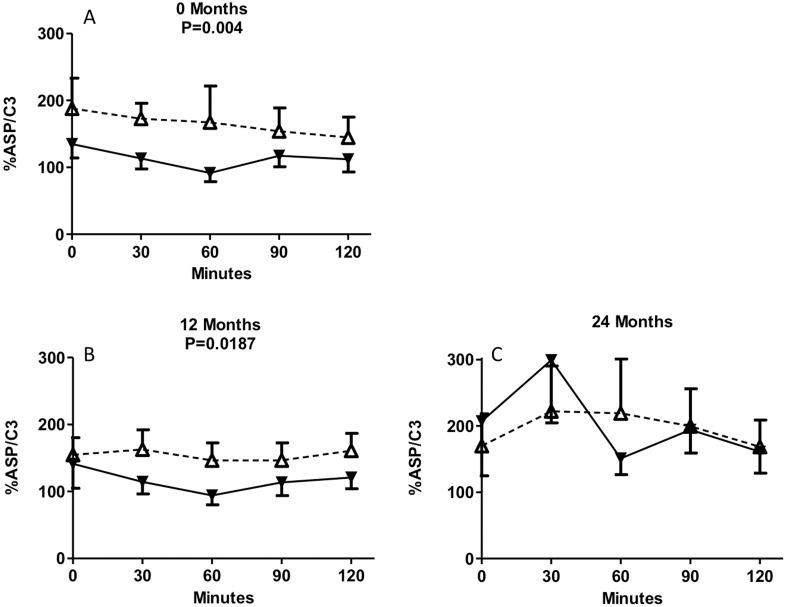
Circulating concentrations of ASP/C3. KPDM subjects with insulin maintenance (white triangles, dotted line) and KPDM subjects with insulin withdrawal (black triangles, solid line) were evaluated at baseline and follow-up. Following a standard mixed meal, ASP was measured from 0 to 120 minutes at baseline (A), and 12 months (B). Results are expressed as mean ± S.E.M; data are analyzed by two-way ANOVA with Bonferroni post-tests as described in methods.

As C3 is the precursor to ASP, the ratio of %ASP/C3 was evaluated. The mean levels of %ASP/C3 during the MMTT were significantly decreased in insulin-withdrawal patients at baseline (P = 0.004) and 12 months (P = 0.0187) compared to insulin-maintenance patients (Figure 6A and B).

## Discussion

In recent years, KPDM appears to be increasingly diagnosed in various populations worldwide. While some studies have reported clinical characteristics and factors associated with insulin discontinuation in patients with KPDM [6], [8], [11], the underlying mechanisms remain unknown. To our knowledge, this is the first study examining serum adipokines and lipid metabolic parameters in KPDM patients.

The present study was performed to evaluate potential changes and differences in lipids, C3 and ASP in relation to a mixed-meal tolerance test following a ketotic episode in KPDM subjects with comparison to T1DM patients. Furthermore, according to insulin discontinuation during the follow-up treatment period, KPDM patients were divided into two groups: KPDM subjects with insulin-maintenance, and those with insulin-withdrawal, with comparison during a 2-year follow-up. Firstly, we observed that during the MMTT, KPDM patients showed elevated serum TG and C3 as well as a decreased conversion of C3 to ASP compared to T1DM subjects. Secondly, we showed higher C3 levels, as well as lower ASP and NEFA levels in KPDM insulin-withdrawal patients. Finally, we demonstrated that NEFA concentrations decreased during the MMTT only in insulin withdrawal patients.

### Clinical characteristics and changes of adipokines during the MMTT in KPDM and T1DM patients at baseline

It is important to consider whether patients presenting with diabetic ketoacidosis have KPDM or T1DM. Correct diagnosis of KPDM enables most cases to be treated successfully with oral agents and insulin to be safely withdrawn over a period of a few months [4], [6]. Patients with KPDM are generally older and more obese [4], [5], [6]. C-peptide measurement may also help physicians in the diagnosis. Our data further corroborated these results.

In the present study, we observed that postprandial triglyceride concentrations in KPDM remained elevated, suggesting delayed clearance of exogenous triglyceride-rich lipoproteins, which may result from reduced lipoprotein lipase activity driven by insulin resistance [18]. Thus, the liberation of NEFA from dietary lipoprotein chylomicron triglyceride might be decreased and this may explain why circulating NEFA concentrations were not higher in KPDM versus T1DM patients, although NEFA concentrations are also determined by the release of intracellular fatty acids.

It has been widely recognized that adipose tissue is not only a storehouse for triglyceride, but is a metabolically active organ producing and secreting proteins, enzymes and hormones [12]. C3 and ASP are two adipokines produced from adipose tissue that regulate triglyceride storage. Elevation of plasma C3 has been linked to coronary artery disease (CAD), obesity and elevated fasting and postprandial triglyceride [19]. The increased BMI in KPDM subjects may partially explain the higher baseline levels of C3 vs. the T1DM patients. ASP is generated through interaction of C3 with factor B and the enzyme adipsin [12]. However, in healthy adults, C3 concentrations are present at 225-fold greater molar concentrations than ASP. Thus, only a relatively small proportion of C3 is converted to circulating ASP [12]. Therefore, ASP concentration will depend not only on production of C3, but also on factors that regulate the conversion of C3 to ASP, and subsequent clearance of ASP. Previous studies have shown increased C3 and ASP in diabetics [12], our data demonstrates significantly higher fasting and postprandial C3 and ASP levels in KPDM subjects compared to T1DM. This may partly be contributed to by the increased fat mass in the KPDM vs the T1DM. However, we show that the values of ASP/C3 during the MMTT in KPDM were significantly decreased when compared to T1DM, which suggests that the conversion of C3 to ASP was decreased in KPDM patients. This may be one of the reasons that we observe high C3 but not comparably increased ASP in KPDM patients. Furthermore, ASP has been shown to stimulate insulin secretion [20], and may contribute to this in KPDM patients, with ASP increased as compensation.

### Changes of adipokines during the MMTT and two-year follow-up in insulin maintenance and insulin withdrawal patients

After resolution of diabetic ketoacidosis and hyperglycemia, exogenous insulin can be safely discontinued in a portion of KPDM patients, and these subjects maintain good glycemic control with diet or with oral hypoglycemic agents [21], [22]. In the present study, fifty-five percent (11 of 20) of KPDM patients discontinued exogenous insulin in the months following their ketotic episode.

Several studies have evaluated clinical characteristics and beta-cell function as predictors of insulin discontinuation in KPDM patients [11], [21], [22]. Maldonado et al [11] considered that obesity was not a predictor of insulin discontinuation in KPDM patients, as there was no BMI difference between insulin-maintenance and insulin-withdrawal patients. However, in another study, KPDM subjects with insulin-withdrawal were more obese than KPDM with insulin-maintenance [6]. In the present study, BMI in insulin-withdrawal subjects was also significantly higher than in insulin-maintenance patients. How the presence of increased BMI may contribute to the potential for insulin withdrawal remains to be determined.

We noticed that NEFA concentrations and NEFA ΔAUC were significantly decreased in insulin-withdrawal patients compared to insulin-maintenance patients over the two years. Circulating NEFAs can be generated either from postprandial dietary lipoproteins through the lipolytic action of lipoprotein lipase, or through adipose tissue release of stored NEFA through the action of intracellular hormone-sensitive lipase [23]. Both increased uptake into adipose tissue (for storage) and decreased output from adipose tissue can contribute to decreased circulating NEFA levels. *In vivo*, postprandial adipose tissue trapping of LPL-generated NEFAs was shown to be close to zero at fasting and close to 100% at 1 h postprandial, decreasing to 10–30% by 6 h [24]. The rate at which NEFAs were taken up by adipocytes is determined by the rate at which they can be resynthesized into intracellular storage triglyceride. ASP has effects on this outcome in adipocytes [16]. In the present study, there were no group differences in triglyceride, and the postprandial changes in triglyceride were relatively moderate, not surprising give the low fat content of the MMTT; however ASP may still be contributing to increased uptake and storage of lipids. On the other hand, postprandial insulin inhibition of hormone-sensitive lipase activity strongly decreases adipose tissue NEFA release [23], and the drop in NEFA in the KPDM insulin-withdrawal group would be evidence of increased insulin sensitivity in this group, while the absence of insulin (which was not provided at the time of MMTT), would explain the absence of insulin-mediated decrease in postprandial NEFA in this group.

We hypothesized that serum C3 and ASP concentrations might be increased during the MMTT because chylomicrons are strong activators of adipocyte C3-ASP production *in vitro* and *in vivo* [25], [26]. A postprandial C3 increment after a fat meal has been shown in healthy subjects, metabolic syndrome patients, CAD patients and in familial combined hyperlipidemia [19], [25], [27]. Nevertheless, we failed to observe significant changes in postprandial C3 in either group, which was similar to other studies [28], [29]. As well, circulating ASP levels did not fluctuate after MMTT in our study [30]. The explanation for this is likely the specific mixed meal composition [30]. In the present MMTT, there was only 14% fat but 65% carbohydrate in the mixed meal. When glucose was added to an oral fat load, the postprandial NEFA response was reduced, and chylomicron-induced activation of C3-ASP was prevented [30]. This may explain why no change in postprandial C3 and ASP concentrations were seen in our study. Further, as demonstrated previously, C3 and ASP concentrations can be elevated locally within the adipose tissue milieu, without any overt changes in the circulation [31], [32].

Although there was no change during MMTT, the C3 concentrations were significantly higher in the insulin-withdrawal group than in the insulin-maintenance patients. This elevated C3 might be related to higher BMI in the KPDM insulin-withdrawal patients. Interestingly, in spite of higher C3, the levels of ASP and the values of ASP/C3 ratio were significantly lower in insulin withdrawal patients than in insulin maintenance patients. Data from this study support the concept that the decreased ASP is consistent with better ASP sensitivity and might contribute to the improved insulin sensitivity in insulin withdrawal patients. Conversely, the lower C3, yet higher ASP and NEFA levels are consistent with ASP resistance in the KPDM insulin maintenance group. However, this also raises the question of what mediated these changes in ASP levels. As discussed above, ASP concentration can be regulated at several levels and mediated by multiple factors, including its precursor C3, the enzymes factor B and adipsin which are involved in C3 cleavage, chylomicrons, hormones, adipokines and drugs [31], [33], [34]. There was a decreased conversion rate of C3 to ASP in KPDM insulin-withdrawal patients, based on decreased ASP/C3 ratio. Interestingly, all the insulin-withdrawal subjects received metformin treatment after discontinuation of exogenous insulin, and *in vitro*, metformin effectively decreases ASP production, without a change in C3 secretion [35]. Although metformin was withdrawn at least 12 hours prior to MMTT, and would not likely have a direct postprandial effect during MMTT, nonetheless a residual effect on ASP production prior to MMTT may still remain.

On the other hand, while ASP levels are increased in a number of metabolic disorders associated with obesity [12], [36], these do not always follow hand in hand. Obesity is not an essential feature of elevated ASP levels, as ASP is still increased in lean subjects with T2DM [37], suggesting that elevated ASP may be a compensatory increase associated with adipose tissue dysfunction or insulin resistance [38]. For the same reason, levels of ASP in KPDM insulin-maintenance patients were elevated in spite of lower BMI. An increased level of ASP might indicate an ASP resistant state [39]. At the cellular level, ASP binds to its cell surface receptor C5L2 to stimulate fatty acid esterification and glucose transport [40]. In ASP deficient (C3 knockout) mice and C5L2 (ASP receptor) knockout mice, the absence of a functional ASP-C5L2 pathway leads to delayed postprandial triglyceride clearance after administration of a fat load [41], [42]. We hypothesize that in the presence of ASP resistance, the ASP-C5L2 pathway is inhibited in KPDM insulin-maintenance patients, and subsequently causes delayed NEFA and triglyceride clearance. This hypothesis is supported by *in vitro* data showing that cells from subjects with high ASP levels have a reduced specific binding and response to ASP [43]. On the other hand, high circulating NEFA concentrations lead to a decrease in mRNA expression and cell surface levels of C5L2 [44]. This would aggravate ASP resistance and delayed NEFA clearance in insulin maintenance patients. Increased circulating NEFA also directly contribute to insulin resistance [45].

In post-bariatric surgery obese subjects, an acute decrease in ASP is predictive of a subsequent increase in insulin sensitivity even before weight loss [46]. We would speculate that KPDM subjects with some maintenance of β-cell function (indicated by C-peptide levels) and a low ASP or ASP/C3 ratio, in spite of higher BMI, would be the most likely candidates for insulin withdrawal.

## Conclusion

In summary, these results demonstrate differences in lipid metabolism as well as in C3 and ASP levels between KPDM and T1DM patients and also between KPDM patients with insulin-maintenance vs. insulin-withdrawal. We demonstrate a decreased conversion of C3 to ASP and delayed triglyceride clearance in KPDM patients vs T1DM. We also demonstrate improved insulin sensitivity in KPDM insulin-withdrawal patients and suggest an improvement in ASP sensitivity in spite of higher BMI.
